# Total Training Volume and Muscle Soreness Parameters Performing Agonist or Antagonist Foam Rolling between Sets

**DOI:** 10.3390/sports9050057

**Published:** 2021-04-29

**Authors:** Haroldo Gualter Santana, Bruno Lara, Filipe Canuto Almeida da Silva, Pedro Medina Eiras, Gabriel Andrade Paz, Jeffrey M. Willardson, Humberto Miranda

**Affiliations:** 1 LADTEF—Performance, Training, and Physical Exercise Laboratory, Federal University of Rio de Janeiro, Rio de Janeiro 21941-599, Brazil; gabriel.andrade.paz@gmail.com (G.A.P.); humbertomirandaufrj@gmail.com (H.M.); 2 School of Physical Education and Sports, Federal University of Rio de Janeiro, Rio de Janeiro 21941-599, Brazil; 3 Lato Sensu Postgraduate Program in Strength Training, Federal University of Rio de Janeiro, Rio de Janeiro 21941-599, Brazil; ef.brunolara@yahoo.com.br (B.L.); filipe.canuto23@gmail.com (F.C.A.d.S.); pedro16medina@gmail.com (P.M.E.); 4 Biodesp Institute, Rio de Janeiro 21020-170, Brazil; 5 Health and Human Performance Department, Montana State University Billings, Billings, MT 59101, USA; jeffrey.willardson@msubillings.edu

**Keywords:** resistance training, self-myofascial release, foam rolling

## Abstract

Background: Foam rolling (FR) has become very popular in recent years; however, the practice of FR between sets of resistance training (RT) for the lower limbs needs further examination. Purpose: Therefore, the purpose of the present study was to examine the effect of FR for the agonists (quadriceps) and antagonists (hamstrings) between multiple sets of the leg extension on repetition maximum performance (RM), fatigue resistance index (FRI), and muscle soreness (MS). Study design: Quasi-experimental clinical trial. Methods: Twenty trained men participated in this study (30.35 ± 6.56 years, 1.77 ± 0.05 cm, 87.70 ± 7.6 kg) and attended seven sessions with 48 h between sessions, (one familiarization session; two 10-RM test and retest sessions; and four experimental sessions). The four experimental sessions were performed in random order and included: agonist foam rolling (AFR), antagonist foam rolling (ANTFR), agonist/antagonist foam rolling (A/ANTFR), and traditional control (TP, without foam rolling). All sessions consisted of three sets for maximal repetitions with a 10-RM load for the leg extension. In the AFR and ANTFR sessions, there was a 120 s rest interval between sets, during which FR was done for the agonists or antagonists, respectively. In the A/ANTFR protocol, there was a 120 s rest interval between sets, during which FR was done for the agonists and antagonists. In the traditional protocol (TP), there was a 120 s passive rest interval between sets. Results: Regarding the total training volume (TTV), significant differences were noted between sessions (F_3,57_ = 11.014; *p* = 0.0001). The AFR, ANTFR, and A/ANTFR sessions had significantly higher TTV versus the TP (*p* < 0.05). Regarding the FRI, significant differences were noted between sessions (F_3,57_ = 2917, *p* = 0.042). A significantly higher fatigue index was shown for the ANTFR and AFR sessions versus the TP (*p* < 0.05). Regarding the total number of repetitions, significant differences were noted between sessions (F_3,57_ = 11.086, *p* = 0.0001). The total number of repetitions was significantly higher in the A/ANTFR, ANTFR, and AFR versus the TP session (*p* < 0.05). MS was significantly lower in the A/ANTFR, ANTFR, and AFR sessions versus the TP session (*p* < 0.05). Conclusion: In conclusion, foam rolling between sets for the agonist or antagonist separately or in succession, resulted in greater neuromuscular performance and higher fatigue indices, as well as reducing the perception of acute muscle soreness.

## 1. Introduction

Foam rolling (FR) is a self-massage technique that, in recent years has become common practice and recommended by coaches and resistance training practitioners (RT). This technique has the main characteristic of using body weight to exert pressure onto specific myofascial regions [1]. Previous studies suggest that the acute benefits of FR are related to an increased range of motion [2], reduced muscle soreness [3], improved performance [4], improved endothelial function [5], blood flow [6], and recovery [4]. The literature proposes several mechanisms (global and local) that support the use of FR. The probable global mechanisms suggest that the results obtained with FR are a consequence of stimulating central pain modulators and significantly reducing parasympathetic activity [7]. Furthermore, local mechanisms consist of changes in thixotropic properties and reduced afferent excitability [7].

The popularity of FR is justified as a low-cost modality that can conveniently be performed pre- and post-exercise. Several studies have observed the effect of FR on performance and recovery; that is, the use of FR as a component of warming up or as a strategy for post-exercise recovery [8,9,10]. Additionally, a systematic review by Latella et al. [11] examined interesting training strategies that included FR. Some hypotheses on FR mechanisms have postulated that neurophysiological interactions in acute pain and removal of metabolites or changes in coactivation (agonist-antagonist relationship) could influence performance and/or recovery. The mechanisms that explain the beneficial effects of FR on muscle pain remain unclear, however, some authors speculate that these effects are influenced by neurological central mechanisms, rather than from local origin [12].

Nascimento da Silva et al. [13] investigated the effect of 60 s of FR between sets for the quadriceps (agonists) on the performance of the leg extension in ten trained individuals. There was not a significant increase or reduction in performance versus a traditional protocol with passive rest interval between sets. However, the study did not involve FR of the antagonists, nor did it investigate acute muscle soreness and fatigue that can influence performance.

Additionally, other studies have examined the effect of FR during the recovery interval between high-intensity tasks. D’Amico and Paolone [14] did not find any benefit from FR of the lower limbs versus a passive interval between two 800 m runs. However, Monteiro et al. [15] reported a reduction in repetition performance in the leg extension when using FR between sets for the posterior thigh region (antagonists) in women. Nevertheless, more research is needed to establish the effectiveness of FR for the agonist/antagonist muscles. In addition, small methodological changes in the application of this technique can directly influence performance, requiring a wide observation of these effects for practical, safe, and efficient application.

Indeed, even with the mixed research results and the absence of a consensus on effectiveness, FR is widely used in training centers, gyms to improve performance. Therefore, the results of the present study may provide information for professionals and practitioners on the best strategy to apply FR between resistance training sets. With that in mind, the purpose of the present study was to examine the effect of FR for the agonists (quadriceps) and antagonists (hamstrings) between multiple sets of the leg extension on repetition maximum performance (RM), fatigue resistance index (FRI) and muscle soreness (MS). As a hypothesis, we expected an improvement in performance with the use of FR between sets compared to the passive rest interval.

## 2. Methods

### 2.1. Subjects

Twenty recreationally trained men (30.35 ± 6.56 years, 177 ± 0.05 cm, 87.70 ± 7.6 kg) participated in this study. The *n* equal to 20 was determined through a statistical calculation appropriate to the characteristics of the present study [16,17]. The following parameters were adopted: (Effect Size = 0.45; β = 0.95; α = 0.05). As inclusion criteria, the following attributes were adopted: (a) resistance training experience of at least one year; (b) frequency of at least three times a week and 50 to 60 min per session; (c) use of loads from 8 to 12 RM in the training routine. The exclusion criteria included: (a) presence of injuries or osteoarticular limitations that would be compromised by the execution of required movements; (b) use of anabolic steroids or dietary supplements that could affect performance in the testing sessions; (c) had a positive response on the Physical Activity Readiness Questionnaire (PAR-Q).

The subjects signed an informed consent form in accordance with the Declaration of Helsinki and the protocol was fully approved by a University Clinical Research Ethics Committee before the beginning of the evaluations, through the process: (CAAE: 63129616.0.0000.5257). The Physical Activity Readiness Questionnaire (PAR-Q) was received from all subjects after a detailed explanation of the benefits and risks involved with the present study. Subjects were instructed on proper hydration and to avoid any exercise during the testing period and to maintain eating habits.

### 2.2. Procedures

**Figure 1:**
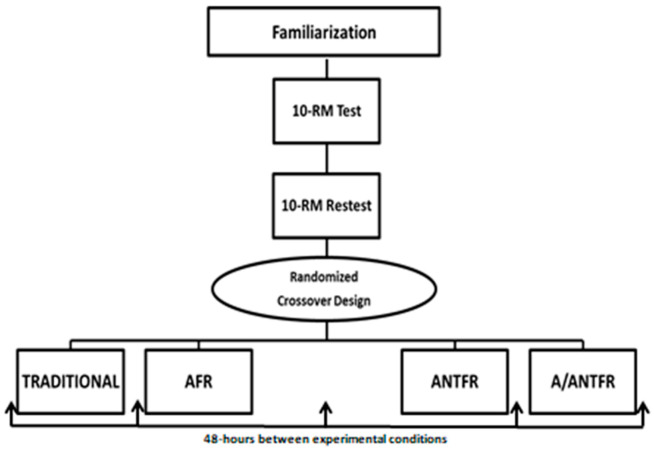
Experimental design; Traditional: traditional protocol; AFR: agonist foam rolling; ANTFR: antagonist foam rolling; A/ANTFR: agonist/antagonist foam rolling.

A randomized crossover clinical trial was conducted. Seven visits were made; the first being for the acquisition of anthropometric data, familiarization with the standardized execution of the exercises, and instructions on the data collection procedures. In addition, all were instructed to perform a 30 s set of FR on the quadriceps and hamstrings to standardize the technique. The next two sessions were intended to test and retest the 10 RM load for the leg extension. In order to verify the total training volume (TTV), total repetitions (TR), fatigue resistance index (FRI), and muscle soreness (MS), the subjects participated in four experimental sessions through random entry in sessions separated by 48 h (Figure 1):

- Agonist foam rolling (AFR): The agonists were the quadriceps muscles. Three sets were performed for maximum repetitions with a 10-RM load in the leg extension exercise, with the performance of 60 s FR for each leg separately between sets [3], totaling 120 s of rest interval.
- Antagonist foam rolling (ANTFR): The antagonists were the hamstrings muscles. Three sets were performed for maximum repetitions with a 10-RM load in the leg extension exercise, with the performance of 60 s FR in each leg separately between sets [3], totaling 120 s of rest interval.
- Agonist/antagonist foam rolling (A/ANTFR): Three sets were performed for maximum repetitions with a 10-RM load in the leg extension exercise, with the performance of 30 s FR in each leg separately between sets [3] for the agonists (quadriceps) and antagonists (hamstrings), totaling 120 s of rest interval.
- Traditional protocol (TP): Three sets were performed for maximum repetitions with a 10-RM load in the leg extension exercise, with 120 s of passive rest interval.

FR was performed at a rate of 60 bpm using a metronome (metronome beats–Sotnekick, version 4.6.0). The metronome was used to standardize the speed at which individuals performed foam rolling. All sessions consisted of three sets of leg extension and the load was kept constant at an absolute 10-RM. Before the experimental sessions, the same warm-up procedure used in the 10-RM test was performed. The recovery interval of 120 s was adopted following recommendations regarding RT [18] and FR application [19]. All subjects were instructed not to perform any type of training during the study period.

#### 2.2.1. Ten Repetition Loads Determination

The loads for 10-RM were determined for each individual in the exercise leg extension (LE) (Leg Extension Machine, Technogym, made in Italy). The 10-RM test was performed following the protocol proposed by Paz et al. [20] and Miranda et al. [21]; The initial load was estimated according to the weight commonly used during resistance training sessions. The objective of the 10-RM test was to carry out 10 consecutive repetitions at a higher load. If the subject did not accomplish a 10-RM in the first attempt, the weight was adjusted by 4–10 kg, and a minimum 5-min rest period was given before the next attempt. Only three trials were allowed per testing session. In the LE, the individual was positioned seated, hips and knees flexed at approximately 90°. During the concentric phase, they performed the complete extension of the knees keeping the ankle in the neutral position. During the eccentric phase, the individual controlled the knee flexion movement until returning to the initial position. No cadence control was adopted. The following strategies were adopted to reduce the margin of error in the data collection procedures [22,23]: (a) standardized instructions were given before the tests such that the person being tested would be aware of the entire routine involved in the data collection; (b) the individual being tested was instructed on the proper exercise execution; (c) all subjects were given standardized verbal encouragement throughout the tests, and (d) all tests were conducted at the same time of the day for every session.

Leg extension position: The range of motion of the concentric phase was between 90° of flexion and 20° of extension and the range of motion of the eccentric phase was between 20° extension and 90° flexion. The chair support was adjusted according to the axis of the machine in relation to the knee joint, and the distal support just above the tibial malleolus. The feet were kept in dorsiflexion. Positioning was recorded for each individual and standardized in all sessions.

#### 2.2.2. Foam Rolling

**Figure 2:**
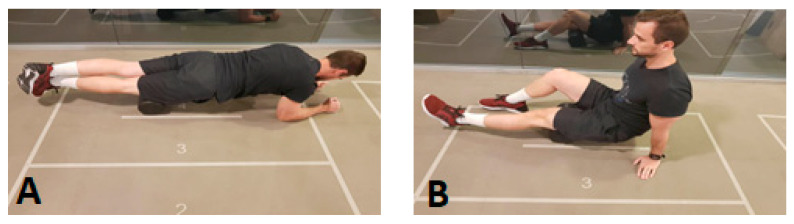
(**A**) Foam rolling (FR) in the quadriceps; (**B**) FR in the hamstrings.

To perform the FR, a high-density foam roller was used (Mormaii, Brazil). FR for the quadriceps was performed in the region between the apex of the patella and the antero-superior iliac spine (Figure 2A); FR for the hamstrings was performed in the region determined between the gluteal fold and the popliteal region (Figure 2B). Subjects were asked to exert maximum pressure on the FR.

#### 2.2.3. Muscle Soreness

A 10 (ten)-point Likert scale was adapted to assess muscle soreness, with seven response options immediately after the end of the training session. Subjects answered the question that best described their subjective sensation of muscle soreness after palpating the regions (anterior and posterior thigh) submitted to intervention, using the following scale: 0 (zero) (no muscle soreness) 2.5 (undefined muscle soreness, occasional soreness), 4 (mild muscle soreness), 5.5 (moderate muscle soreness), 7 (constant muscle soreness, sore feeling), 8.5 (strong muscle soreness), 10 (unbearable muscle soreness sensation).

#### 2.2.4. Fatigue Resistance Index

The fatigue resistance index, associated with a reduction in repetitions in the training session, was calculated according to the following formula: FRI = (third set/first set) × 100; where higher percentage values (%) indicate greater resistance to fatigue [24].

### 2.3. Statistical Analyses

Descriptive statistics were applied in order to characterize the sample, using the mean, median, and standard deviation as measures of central tendency and dispersion respectively. The statistical treatment was performed using SPSS software version 18.0 (Chicago, IL, USA). The statistical analysis was performed initially using the Shapiro–Wilk test of normality and homoscedasticity. In sequence, a one-way ANOVA with repeated measures was used to determine if there was a significant difference between sessions in TR, TTV, and FRI. The equation (number of sets x number of repetitions x load) was used to calculate the TTV for the leg extension [25]. The TR was calculated as the sum of repetitions during the three sets of leg extension. Post-hoc tests using the Bonferroni correction were employed. The Friedman nonparametric test was applied to compare muscle soreness between sessions. Additionally, to determine the magnitude of differences, effect size statistics (ES; the difference between pre-test and post-test scores divided by the pre-test standard deviation) were calculated for the LE for all sessions. The magnitude of the ES was interpreted using the scale proposed by Rhea [26] for recreationally trained individuals, in which an ES lower than 0.35, 0.35–0.80, 0.80–1.5, and higher than 1.5 were referred to as trivial, small, moderate, and large effects respectively. The level of significance was set as *p* < 0.05.

## 3. Results

The intraclass correlation coefficient for the 10RM test-retest was 0.855 for the leg extension. With respect to TTV, there was a significant difference between the sessions (F_3,57_ = 11.014; *p* = 0.0001; Table 1). The TTV was significantly higher in sessions A/ANTFR (*p* = 0.001), ANTFR (*p* = 0.007) and AFR (*p* = 0.025) versus the TP session. With respect to TR, there was a significant difference between the sessions (F_3,57_ =11.086, *p* = 0.0001; Table 1). The TR were significantly higher in sessions A/ANTFR (*p* = 0.0001), ANTFR (*p* = 0.008) and AFR (*p* = 0.025) versus the TP session.

With respect to the fatigue resistance index, there was a significant difference between the sessions (F_3,57_ = 2917, *p* = 0.042; Table 1). The fatigue resistance index was significantly higher in sessions ANTFR (*p* = 0.010) and AFR (*p* = 0.055) versus the TP session. However, the post hoc test showed no significant difference between the A/ANTFR protocol versus the TP session.

With respect to muscle soreness, the Friedman test indicated a significant difference between sessions [x^2^ (3) = 33.526; *p* = 0.001]. Muscle soreness (see Table 1) was significantly less in sessions A/ANTFR (*p* = 0.0001), ANTFR (*p* = 0.001) and AFR (*p* = 0.011) versus the TP session. Similar results were verified when comparing the A/ANTFR session versus the ANTFR (*p* = 0.019) and AFR (*p* = 0.003) sessions.

The ES was described in Table 2. A small ES was observed for the AFR and moderate ES for the ANTFR and A/ANTFR in relation to the TP. With respect to TTV, there was a small ES in the AFR and ANTFR and moderate ES in A/ANTFR. With respect to FRI, there was a moderate ES for all intervention sessions.

## 4. Discussion

Foam rolling between sets for the agonist or antagonist separately or in succession resulted in greater neuromuscular performance and higher fatigue indices, as well as reducing the perception of acute muscle soreness, confirming the initial hypothesis. Additionally, the effect size observed for TTV, TR, and FI reinforces the benefits of the interventions performed versus the TP.

When comparing the outcomes of the present study with the previous literature, there have been mixed results. Three studies by Monteiro et al. [15,27,28], investigated the effect of FR between sets in 25 recreationally active women. Regarding the use of FR between sets for the antagonist muscles during the leg extension exercise, Monteiro et al. [15] found a negative effect on repetition performance over three sets, in interventions that consisted of 60 and 120 s of FR, respectively. These results contrast with the present study with regard to total repetitions and total training volume since we observed an improvement in performance in all interventions with FR between sets versus a passive rest. A key factor to account for differences between studies might be in the study by Monteiro et al. [15], both experimental conditions with FR (60 and 120 s) between sets were performed in the same training session; whereas, in the present study, a 48-h interval was adopted between sessions in order to provide greater recovery for subjects. The positive results in repetition performance exhibited by foam rolling might be due to improved blood flow and restoration of intramuscular pH to enable greater consistency in contractile performance.

Furthermore, Nascimento da Silva et al. [13], did not find significant differences in repetition performance with 70% of 1RM for the dominant limb in the leg extension, using 60 s of foam rolling for the quadriceps (agonists) between sets. However, the performance improvements in TTV and TR with foam rolling in the present study might be due to subjects’ prior experience with the FR technique. Evidence suggests that individuals with experience in FR may have reduced perceptions of muscle soreness, that is, a positive change in pain thresholds as a result of mechanoreceptor and chemoreceptor interactions present in the connective and muscular tissue [29]. Additionally, it is suggested that a central pain modulator is responsible for changes in pain perception after using FR [12].

A higher fatigue index was observed in the AFR and ANTFR sessions versus the TP. However, the A/ANTFR protocol, even if not significantly different, was higher than the TP and had a moderate effect size. The results contrast with the study by Monteiro and Neto [28], who observed a negative dose-response effect in the FRI after foam rolling between sets for the agonist muscles in the leg extension exercise. Sessions were performed with 60, 90, and 120 s of FR, and the IF was significantly reduced in all interventions with FR adopting a relationship with the time of exposure to FR. Interestingly, in the study by Monteiro and Neto [28] cadence control was adopted, which can influence the time under tension, fatigue, and strength of subjects. The use of cadence control in repetition maximum sets may limit the ability to compare studies that did not report using cadence control. The mechanisms underlying the beneficial results of the present study on fatigue tolerance may be related to increased blood flow [6] and consequently optimization of the removal of fatigue-inducing metabolites from localized muscle contractions in the quadriceps. Additionally, thixotropic changes in the myofascial complex (muscle + fascia) from foam rolling may help restore the mechanical properties of the tissue [19].

The TTV and TR in the present study showed significant benefits with the use of FR between sets. Similarly, the FRI and muscle soreness expressed similar outcomes. It is possible to infer that the increase in fatigue tolerance due to the FR between sets allowed for greater repetition performance and, consequently, a greater TTV. Increased fatigue tolerance associated with low pain rates can provide additional benefits such as reducing injury risk factors and maintaining movement quality. The results presented in the current study suggest that FR was efficient in reducing the perception of muscle soreness after the training session; several mechanisms that clarify the perception of muscle soreness after exercise, such as damage to connective tissue and inflammation [29].

From this perspective, foam rolling between sets can effectively increase tolerance to stretching, which can be an important mechanism in reducing the perception of muscle soreness. Previous studies that reported negative effects when foam rolling between sets on performance show methodological differences that were mentioned throughout the present narrative [15,27,28]. Additionally, the difference between genders is a factor that deserves attention, since sensitivity and pain tolerance may prompt different subtle differences in foam rolling technique that could affect performance outcomes, whether positive or negative [30,31]. It is worth mentioning that these mechanisms need to be investigated to allow more robust inferences.

As a limitation of the present study, we can highlight the performance benefits of foam rolling for only the leg. Therefore, it is suggested to carry out studies with a complete session of exercises, as well as conducting a study under this perspective of intervention with foam rolling between sets using additional tools for monitoring fatigue. Additionally, we note the absence of a sham protocol to avoid a possible placebo/nocebo effect. However, it is noteworthy that these limitations are inherent to studies conducted with FR, since, to date, we have not identified studies that have performed interventions with FR and adopted a sham group in the methodology. It is worth mentioning the ecological validity of the present study since the experimental sessions were designed to simulate the training environment of the vast majority of coaches and practitioners when adopting foam rolling.

## 5. Conclusions

In conclusion, foam rolling between sets for the agonist or antagonist separately or in succession resulted in greater neuromuscular performance and higher fatigue indices, as well as reducing the perception of acute muscle soreness. It is worth to mention that the performance benefits were verified only in the muscles submitted to the intervention. Therefore, it is recommended that professionals and practitioners use foam rolling between sets as a strategy to optimize performance in the lower limbs.

## Figures and Tables

**Table 1:** Neuromuscular variables. Mean ± SD (95% confidence intervals) and median muscle soreness.

	Total Repetitions	Total Training Volume (kg)	Fatigue Resistance Index (%)	Muscle SORENSS
TP	40.65 ± 7.05 (37.350–43.950)	3833 ± 757 (3478–4187)	89.61 (8.25)	5 #
AFR	44.70 ± 6.93 * (41.454–47.946)	4205 ± 724 * (3866–4544)	98.85 (18.77) *	4 *#
ANTFR	46.40 ± 6.07 * (43.558–49.242)	4357 ± 592 * (4080–4634)	99.15 (14.15) *	4 *#
A/ANTFR	47.95 ± 5.92 * (45.177–50.723)	4509 ± 641 * (4209–4809)	96.44 (16.27)	2 *

* Significant difference versus the traditional protocol (*p* ≤ 0.05). # Significant difference versus the agonist-antagonist session. TP: traditional protocol; AFR: agonist foam rolling; ANTFR: antagonist foam rolling; A/ANTFR: agonist and antagonist foam rolling.

**Table 2:** Effect size and classification of neuromuscular variables between foam rolling sessions versus traditional.

	Total Repetitions	Total Training Volume	Fatigue Resistance Index
AFR	0.57 (Small)	0.49 (Small)	1.12 (Moderate)
ANTFR	0.82 (Moderate)	0.69 (Small)	1.16 (Moderate)
A/ANTFR	1.04 (Moderate)	0.89 (Moderate)	0.83 (Moderate)

AFR: agonist foam rolling; ANTFR: antagonist foam rolling; A/ANTFR: agonist and antagonist foam rolling.
